# Serial dependence improves performance and biases confidence-based decisions

**DOI:** 10.1167/jov.23.7.5

**Published:** 2023-07-06

**Authors:** Paula A. Maldonado Moscoso, David C. Burr, Guido Marco Cicchini

**Affiliations:** 1 CNR, Institute of Neuroscience, Pisa, Italy; 2 CIMEC – Center for Mind/Brain sciences, University of Trento, Italy; 3 Department of Neuroscience, Psychology, Pharmacology and Child Health, University of Florence, Firenze, Italy; 4 CNR, Institute of Neuroscience, Pisa, Italy

**Keywords:** serial dependence, perceptual decision, visual perception, sequential effects

## Abstract

Perception depends on both the current sensory input and on the preceding stimuli history, a mechanism referred to as serial dependence (SD). One interesting, and somewhat controversial, question is whether serial dependence originates at the perceptual stage, which should lead to a sensory improvement, or at a subsequent decisional stage, causing solely a bias. Here, we studied the effects of SD in a novel manner by leveraging on the human capacity to spontaneously assess the quality of sensory information. Two noisy-oriented Gabor stimuli were simultaneously presented along with two bars of the same orientation as the Gabor stimuli. Participants were asked to choose which Gabor stimulus to judge and then make a forced-choice judgment of its orientation by selecting the appropriate response bar. On all trials, one of the Gabor stimuli had the same orientation as the Gabor in the same position on the previous trial. We explored whether continuity in orientation and position affected choice and accuracy. Results show that continuity of orientation leads to a persistent (up to four back) accuracy advantage and a higher preference in the selection of stimuli with the same orientation, and this advantage accumulates over trials. In contrast, analysis of the continuity of the selected position indicated that participants had a strong tendency to choose stimuli in the same position, but this behavior did not lead to an improvement in accuracy.

## Introduction

We are brought to think that our perceptual experience of the world is always truthful. However, as experienced during many illusions, such as Gregory's (1997) “hollow mask illusion” (https://michaelbach.de/ot/fcs-hollowFace/), perception may be biased by spatial and temporal context. Studies investigating serial dependence (SD; Cicchini, Mikellidou, & Burr, in press) showed that individual perceptual judgments of the current stimulus tend to incorporate past information even after a long time, robustly influencing current perception.

SD has been shown to be strongest when the sequential objects are nearby in retinal location, showing that it is spatially selective and suggesting that the selectivity is largely retinotopic (Collins, 2019; Corbett, Fischer, & Whitney, 2011). Other groups have found that the selectivity is world rather than retinal centered (Fischer & Whitney, 2014; Mikellidou, Cicchini, & Burr, 2021). Previous studies also found that multiple continuity fields operate simultaneously on individual objects within multiple-object scenes, integrating object representations and ensemble representations with previous history (Collins, 2022). SD effects have been observed not only for orientation (Fischer & Whitney, 2014; Liberman, Zhang, & Whitney, 2016; Manassi, Liberman, Chaney, & Whitney, 2017), but also for numerosity (Cicchini, Anobile, & Burr, 2014; Corbett et al., 2011), facial gender and expression (Liberman, Fischer, & Whitney, 2014; Xia, Leib, & Whitney, 2016), and even beauty (Kondo, Takahashi, & Watanabe, 2012; Taubert, Van Der Burg, & Alais, 2016). Typically, serial dependence effects have been equated to an attractive bias toward previous stimuli, but repulsive biases have also been described (Fornaciai & Park, 2019b; Fritsche, Spaak, & de Lange, 2020; Moon & Kwon, 2022; Sheehan & Serences, 2022; for reviews, see Cicchini et al., in press; Pascucci et al., 2023).

Although the idea that SD results from the integration of sensory information is theoretically compelling, others have suggested that the SD acts on later decision stages rather than on sensory mechanisms. However, evidence in favor of this idea is not strong. The original report by Fischer and Whitney (2014) provided evidence that SD occurs even when observers are not asked to make a behavioral response, showing that these mechanisms are in part automatic. Likewise, changing the response so participants alternately reproduce the actual orientation and the mirror orientation has little impact on serial effects, demonstrating that the response format is not critical (Cicchini, Mikellidou, & Burr, 2017). A recent study showed that SD occurs only for consciously perceived stimuli, suggesting that the construction of priors (i.e., knowledge accumulated from perceptual history) requires a degree of processing (Kim, Burr, Cicchini, & Alais, 2020). Consistently, orientation SD occurs within visual stimuli of different format, suggesting that low-level processes may not be essential (Ceylan, Herzog, & Pascucci, 2021). On the other hand, several studies claimed that positive SD, which is typically measured in reproduction tasks, occurs at a post-perceptual (i.e., working memory and decision making) decisional stage rather than at the level of perception (Bliss, Sun, & D'Esposito, 2017; Fritsche, Mostert, & de Lange, 2017; Stein et al., 2020). However, recent studies show that, although priors are probably constructed at a reasonably high level, past choice is propagated down to lower sensory levels where serial dependence acts (Cicchini, Benedetto, & Burr, 2021; Collins, 2020; Fornaciai & Park, 2019a). Using functional magnetic resonance imaging (fMRI), St. John-Saaltink, Kok, Lau, and De Lange (2016) showed that the bias toward the previous percept is reflected in activity in the primary visual cortex. In this case, it may reflect the influence of top–down expectations rather than bottom–up accumulation of sensory evidence over trials. In fact, studies have shown that prior expectations can bias sensory representations in the visual cortex (Kok, Failing, & de Lange, 2014; Summerfield & De Lange, 2014). Overall, the evidence suggests that the effects of serial dependence arise and act across multiple brain regions.

Here, we bring fresh evidence to the debate by leveraging on one of the capabilities of human observers, which is to assess spontaneously the quality of their sensory information. A growing body of literature shows that when observers are provided with the possibility to choose which stimulus to judge they improve their performance, indicating that they are aware of the sensory resolution (Barthelmé & Mamassian, 2009).

We adapted Barthelmé and Mamassian's (2009) paradigm, where observers choose which stimulus to judge, to address two related issues: Does the repeated presentation of a stimulus improve discrimination performance, measured as accuracy? Also, does repetition of a feature (orientation) affect observer choice? The first question addresses whether SD, like priming, can improve sensory resolution, and the second addresses whether confidence-based decision mechanisms that select the stimulus act at the site of sensory improvement.

## Methods

Twenty-two participants (11 female; age, 26.3 ± 6.8 years) took part in the experiment and gave informed consent (the research was approved by Commissione per l'Etica della Ricerca, University of Florence), following the tenets of the Declaration of Helsinki. Six participants were excluded from the analysis because their overall accuracy was below 66% of correct trials. All participants except one of the authors (PAMM) were naïve to the purpose of the experiment.

Measurements were made in a quiet room in dim lighting conditions. Stimuli were all generated and presented with Psychtoolbox routines (Brainard, 1997) for MATLAB 2010a (MathWorks, Natick, MA) and displayed on a liquid-crystal display (LCD) with a screen resolution of 1920 × 1080 pixels and refresh rate of 60 Hz, subtending 56° by 32° from the viewing distance of 57 cm.

**Figure 1.**
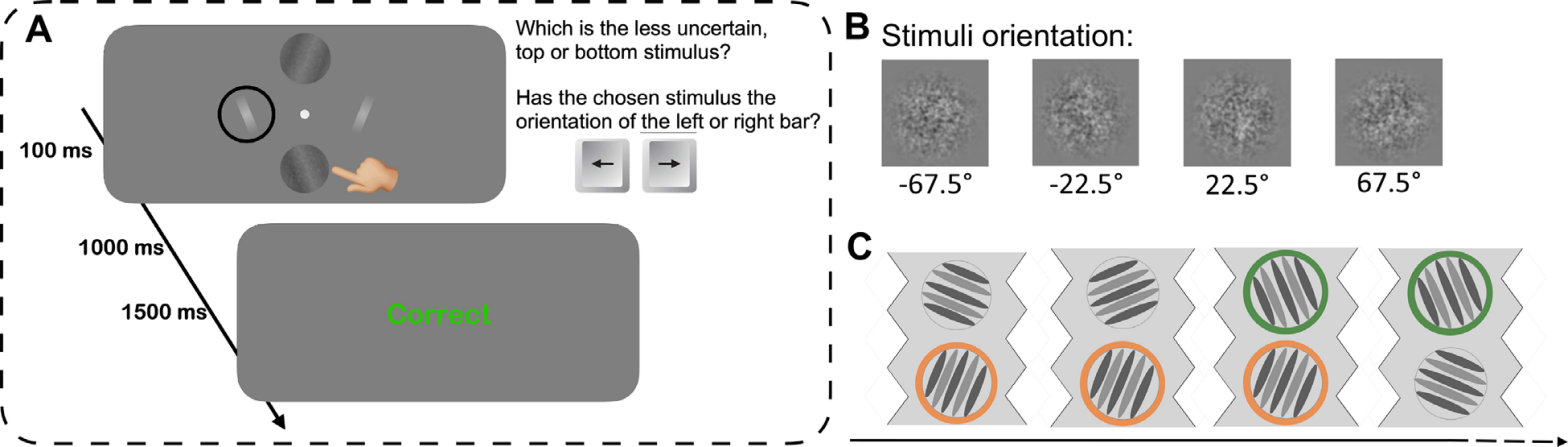
Event sequence and example of stimuli. (**A**) On each trial, two Gabor patches and two white bars were presented, all centered 7.3° from the central fixation point, as shown. Participants selected first the Gabor patch they felt more confident in judging orientation, then which white bar had the same orientation as the selected Gabor patch. After each trial, participants received true visual feedback. (**B**) Example of the four orientations for Gabor patches with 6% of contrast. (**C**) Example of trial sequence. On each trial, one of the Gabor patches had the same orientation and the same position as the Gabor patch in the previous trial. The rings highlight the continuous Gabor patches across trials.

Each trial started with the simultaneous presentation of two Gabor patches and two white bars, straddling the fixation point as shown in Figure 1A. The Gabor stimuli were generated from gratings of 0.33 cycles per degree vignetted by a Gaussian of space constant 5.8°, presented for 100 ms 7.3° above and below fixation. The gratings were embedded in random noise of 16% root-mean-square (RMS) contrast. The contrast of one of the gratings (test stimulus) was selected randomly from the range 1.2% to 6%, and the other was fixed at 3.5% (standard stimulus). The two gratings always differed in orientation from each other by at least 45°. The two white bars (0.8° × 5.8°), positioned 7.3° right and left of fixation, each matched the orientation of one of the two Gabor patches (see Figure 1A).

The technique was like that of Barthelmé and Mamassian (2009): Participants first selected the Gabor patch whose orientation they felt most confident to judge (by pressing the up or down arrow), then indicated which white bar had the same orientation as the chosen Gabor patch (right or left arrow). One second later participants were given on-screen visual feedback about the accuracy of their response.

Gabor patches (and the response bars) were selected from four possible orientations: ±67.5° or ±22.5° (Figure 1B). Crucially, we introduced a constraint in the trial sequences: one of the Gabor patches had the same orientation in the same position as the Gabor patch in the previous trial (Figure 1C), whereas the other Gabor patch was always different from that in the same position of the previous trial.

Each participant completed on average 10 blocks of 100 trials (total 1000 trials). We eliminated trials where the responses on the confidence and orientation task were faster than 0.2 seconds or slower than 3 *SD* of the mean (3.2 seconds and 1.2 seconds, respectively, for the confidence and orientation tasks).

Most analyses were performed on the “aggregate participant” by pooling data from all participants as if they were from a single individual. Analyzing the aggregate participant is a technique in this line of research (Cicchini et al., 2017; Cicchini, Mikellidou, & Burr, 2018; Galluzzi, Benedetto, Cicchini, & Burr, 2022; Kim et al., 2020; Zhang & Alais, 2020). We investigated the history effect up to five previous trials on choice and the resulting accuracy of the orientation task. We also investigated the cumulative history effect (up to five-back) of continuity and discontinuity; these were obtained from isolating data where all the previous trials (from two to five) were either identical to or different from the current one. For each condition, we calculated the baseline probability of choosing the continuous (or discontinuous) stimulus, defined as the number of trials with continuous stimuli divided by the total number of trials. As we forced the continuity between two consecutive trials, the baseline probability of choosing the continuous stimulus at one-back was 0.5 and it was 0.27 at five-back trials. Then, we calculated the number of times participants chose a continuous (or discontinuous) stimulus divided by the total number of trials and subtracted out the corresponding baseline probability in order to obtain the choice bias. We executed the same procedure on shuffled data to obtain the orientation choice bias of the shuffled data.

## Results

### Continuity of orientation

**Figure 2.**
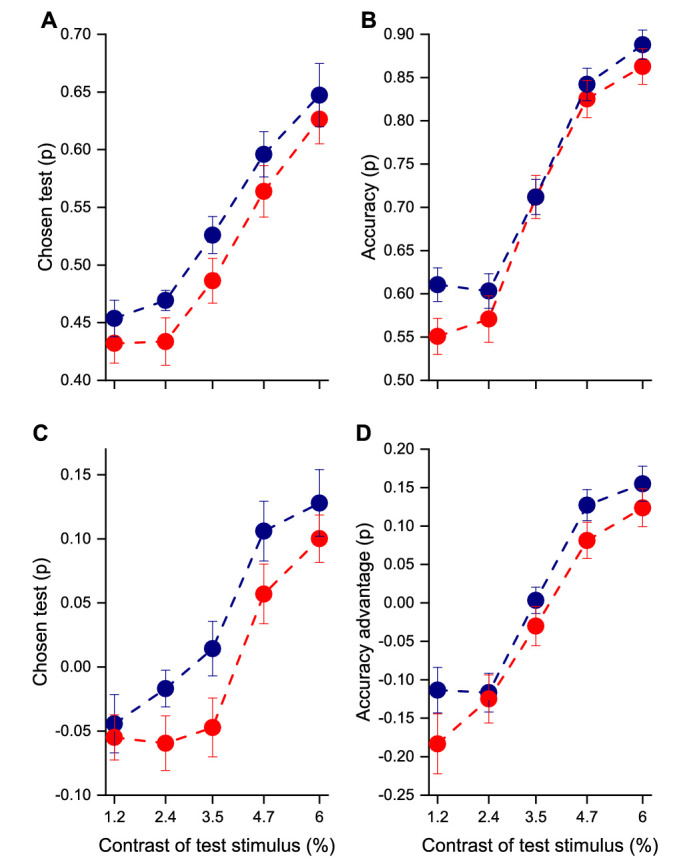
(**A**) Psychophysical functions showing the proportion of times participants chose the test stimulus over the standard. (**B**) Accuracy of the chosen stimulus as a function of the contrast of the test stimulus. (**C**) Psychophysical functions showing the participants’ bias for chosen test over the standard (after subtracting out the effect with shuffled data). (**D**) Accuracy advantage of the chosen stimulus as a function of the contrast of the test stimulus. In all graphs, the blue symbols indicate same orientation and red different orientation. Error bar represents ±1 *SEM.*

We adapted Barthelmé and Mamassian's (2009) dual-response technique to investigate the serial effects of stimulus repetition on both stimulus choice and accuracy of orientation judgments. Participants chose first which Gabor stimulus they felt most confident to judge, then reported the orientation of that stimulus, by selecting the best matching white bar (Figure 1A). Figure 2A plots the proportion of trials the stimulus of test (variable contrast) was chosen over the standard stimulus, as a function of its contrast (the standard stimulus was always 3.5%). The results are plotted separately for trials where the test stimulus orientation was the same as the previous trial (continuous condition: blue symbols) and for those when it was different (discontinuous condition: red symbols). However, for all test contrasts, the chance of choosing the test over the standard was higher when the previous stimulus had the same orientation (continuous curve) than when it did not (discontinuous). We performed a repeated-measures analysis of variance (ANOVA) with contrast (five levels) and condition (continuous and discontinuous) as within-subject factors. The main effect of condition was significant, *F*(1, 15) = 7.93, *p* = 0.013, η^2^ = 0.02, as well as the main effect of contrast, *F*(4, 60) = 24.60, *p* < 0.0001, η^2^ = 0.52. The interaction was not significant, *F*(4, 60) = 0.21, *p* = 0.88, η^2^ = 0.001, suggesting that the continuity effect did not depend on contrast.

Figure 2B plots accuracy (proportion correct) for the two conditions as a function of test contrast. Accuracy also increased with test contrast, as expected. Importantly, for most contrasts the accuracy was higher for the continuous than for the discontinuous stimuli. Repeated-measures ANOVA with contrast (five levels) and condition (continuous and discontinuous) as within-subject factors revealed a significant effect of contrast, *F*(4, 60) = 88.04, *p* < 0.0001, η^2^ = 0.74. Condition was marginally significant, *F*(1, 15) = 3.63, *p* = 0.08, η^2^ = 0.009, and the interaction between contrast and condition was not statistically significant, *F*(4, 60) = 0.84, *p* = 0.51, η^2^ = 0.005.

Analysis of serial effects poses several challenges, as any unwanted correlation between responses and stimuli across trials (for example, if one session were performed with different criteria from the others) may introduce spurious effects. For this reason, we simulated the behavior of a virtual observer who had the same gross statistics as the real observer, but stimuli and responses were shuffled to destroy the strict temporal interrelationship of the real data. Doing so predicted the magnitude of the unwanted spurious effects, enabling us to analyze the temporal order characteristics of the data. Figures 2C and 2D show data from Figures 2A and 2B, plotting the average choice and accuracy biases as a function of test contrast, after subtracting out the shuffled observer responses. Both the choice test bias and the accuracy advantage were higher for continuous than for discontinuous stimuli, *F*(1, 15) = 10.06, *p* = 0.006, η^2^ = 0.03 and *F*(1, 15) = 5.24, *p* = 0.037, η^2^ = 0.015, respectively.

**Figure 3.**
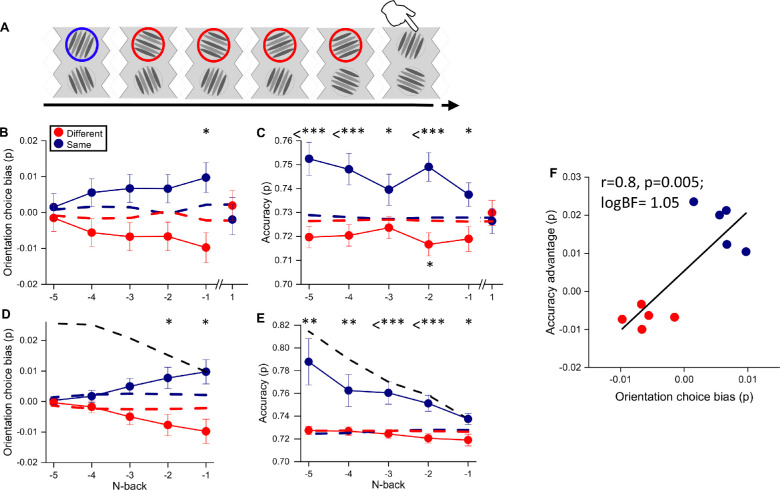
The effect of continuity of orientation on stimulus choice and accuracy. (**A**) Illustration of a typical stimulus sequence showing an example where the orientation was the same five trials back. (**B**) Orientation choice bias separated into trials that were either the same or different *N* trials back. (**C**) Accuracy for the same conditions. (**D**) Orientation choice bias as a function of the cumulative number of *N*-back for trials that were all either the same or different. Black-dashed lines represent the cumulative prediction from the data of Figure 3B. (**E**) Accuracy as a function of the cumulative number of *N*-back for the same conditions. Black-dashed lines represent the cumulative prediction from the data of Figure 3C. (**F**) Accuracy as a function of orientation choice bias, averaged over trials and participants from one-back to five-back. In all graphs, the results of trials classified as different orientations compared with the current selection are represented in red and the same orientations in blue. Blue and red dashed lines show the prediction from shuffled data. Error bar represents ±1 *SEM*. Statistical significance: *p < 0.05; **p < 0.01; ***p < 0.001.

As the results were similar at all contrasts, for all further analyses we pooled the data from all stimuli, irrespective of their contrast. We examined further the effect of stimulus continuity on choice and accuracy for trials as far as five back from the current trial and tested their statistical significance (Figure 3A). Figure 3B plots the choice bias toward the continuous stimulus. The choice bias was defined as the probability of choosing the continuous (or discontinuous) stimulus minus the baseline probability for that condition, defined as the probability of choosing the continuous (or discontinuous) by chance (see Methods). As there were only two choices, the bias for the discontinuous was always equal and opposite to the bias for the continuous stimuli. For the one-back condition, the stimuli of the same orientation as the previous were selected more frequently than the other, by about 1.9% (*Z* = 2.33, *p* = 0.02). The dashed lines show results after shuffling observer responses within a session (average of 10,000 permutations). The difference between the real and shuffled data was significant (*p* < 0.05, bootstrap signed test) for one trial back. The difference remained for up to four trials back but became insignificant (*p* > 0.05). Importantly, there was no effect at all (logBF = −2) of the orientation of the future stimulus, showing that the effect was causal.

Figure 3C plots accuracy separately for when the chosen stimulus orientation was the same or different as the previous *n*th-back trial. For all conditions, accuracy for stimuli with the same orientation was higher than that for different orientations by about 2.4%. In all cases, the difference was significant, often highly so (Figure 3), even for five trials back. Importantly, again there was no significant improvement for choices corresponding to the future trial (logBF = −2). Figures 3D and 3E plot the cumulative effects of orientation similarity. In these plots, the “same orientation” condition refers to all of the previous *N* trials having the same orientation, and “different” refers to no previous trials having the same orientation. Obviously, the number of trials reduced considerably with *N*, from 7795 for *N* = 1 to 768 for five consecutive trials. The bias in choice was significant up to two trials back. Moreover, the improvement in accuracy was greater for the cumulative condition and remained significant even at five-back (all *p* < 0.05). This finding suggests that the benefits of repeating the orientation accumulate over many trials. To compare the two sets of data, we overlaid on Figures 3D and 3E the predictions from the data of Figures 3B and 3C assuming perfect summation (black-dashed lines). The accuracy data are reasonably similar to the perfect summation predictions, but for choice the effect was opposite: the effect diminished over time rather than accumulating. This probably resulted from a reluctance of observers to choose the continuous stimulus when presented in the same location for many consecutive trials. However, when they did, it led to better performance.

To compare the effects of stimulus continuity on stimulus choice and accuracy, Figure 3F plots accuracy against choice bias for all *N*-back conditions, replotting the data of Figures 3B and 3C (maintaining the color code for continuous and discontinuous). The correlation was strong (*r* = 0.8, *p* = 0.005, logBF = 1.05), accounting for 64% of the variance. This shows that stimulus continuity affects both choice and accuracy, in a similar fashion.

**Figure 4.**
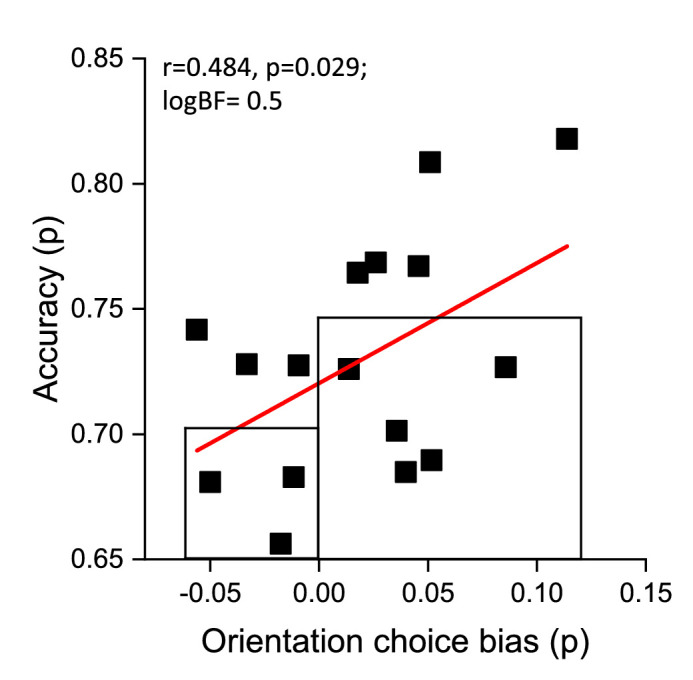
Accuracy as a function of orientation choice bias (tendency to prefer continuous stimuli) for individual participants. The red line shows the best linear fit, which was significant with moderate Bayes factor evidence (*r* = 0.48, *p* = 0.029, logBF = 0.5). The rectangles show the average for participants with negative and positive orientation choice bias. Those with positive choice bias were more accurate, by 4% on average, *t*(14) = 1.92, *p* = 0.038, logBF = 0.4.

We also looked at individual behavior differences to see if the tendency to choose the continuous stimulus led to increased accuracy. Figure 4 plots accuracy against orientation choice bias separately for each participant. We found a positive relationship (*r* = 0.48, *p* = 0.029, logBF = 0.5, one-tailed), showing that participants with a stronger tendency to choose the same orientation (positive values of choice bias) had a better performance compared with those who chose different orientations more times. The rectangles show the average of participants with negative and positive orientation choice bias. Those with positive choice bias were more accurate, by 4% on average, *t*(14) = 1.92, *p* = 0.038, logBF = 0.4.

### Continuity of position

**Figure 5.**
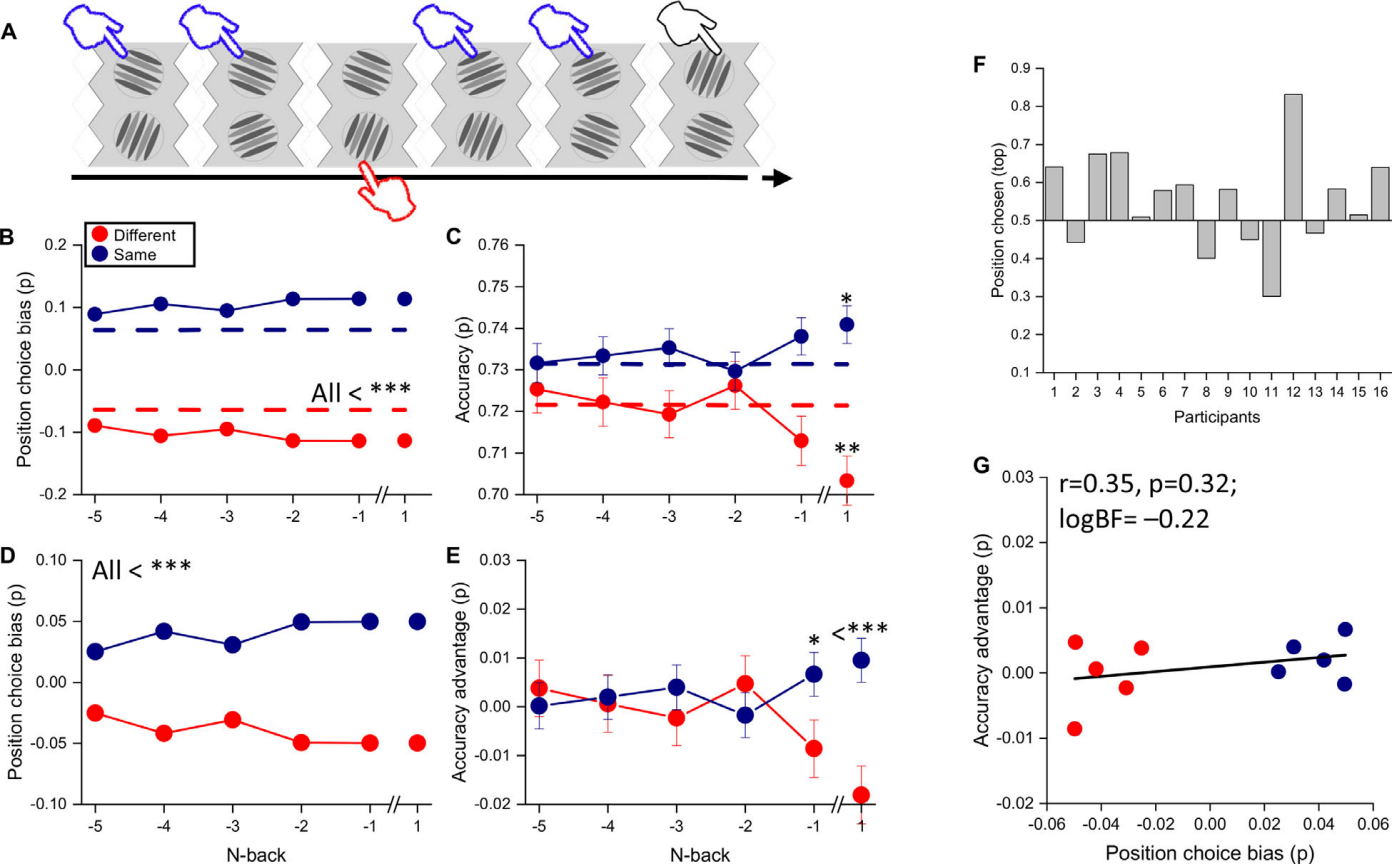
(**A**) Example of classification of the trials with different (red) and same (blue) “physical position selected” compared with the current selection. (**B**) Position choice bias for trials of the same (blue) or different (red) position as the trial *N*-back (raw data). The dashed line shows the average orientation bias with permutated data, where the responses within each session were randomly shuffled (average of 10,000 repetitions). (**C**) Same as (B) but for accuracy. (**D**) Position choice bias (with expanded ordinate) for trials of the same (blue) or different (red) position as the trial *N*-back after subtracting out the effect with permutated data. (**E**) Same as (**D**) but for accuracy advantage. (**F**) Top response bias of all participants. (**G**) Accuracy advantage as a function of orientation choice bias, averaged over trials and participants from one-back to five-back (after removing the effect with permutated data). The black line represents the best-fitting linear regression. Error bar represents ±1 *SEM*. Statistical significance: *p < 0.05; ***p < 0.001.

The complex nature of the paradigm—where participants chose first which stimulus to respond to before judging orientation—allowed us to look for serial effects other than for orientation. For example, is there a tendency to choose the stimulus in the same position as the previous, and does that lead to an improvement of accuracy? To test this, we examined how choice and accuracy were affected by similarity between the current and previous stimuli (see example in Figure 5A). Figure 5B shows the choice bias of the aggregate participant when the observers selected stimuli in the same physical position at the current and *N*-back trials (blue circles) and when observers selected stimuli in different physical positions at the current and *N*-back trials (red circles). The data show that the observers had a very strong tendency to select stimuli in the same position as the last, with a bias of around 10%. The preference was so strong that it remained high even for five items back. This was associated with a slightly higher accuracy for trials on repeated positions (on average, *p* = 0.73) compared with *p* = 0.72 for changed positions.

However, a similar improvement in accuracy was observed for congruency of position with the future stimulus (+1), suggesting that it may be artifactual. Other evidence that the effects may be artifactual is the fact that, when the responses were shuffled within each session, removing temporal continuity (dashed lines of Figures 4B and 4C), the effects on both choice and accuracy remained strong (Figures 4D and 4E). The magnitude of the choice bias with shuffled responses was less than that of the real data (all *Z* > 3.72, *p* < 0.001). However, the accuracy advantage remained also for future trials, showing that the effects were not causal. Remarkably, the improvement for future trials (∆*P* = 0.95%, *Z* = 3.71, *p* < 0.001) was stronger than for one-back (∆*P* = 0.66%, *Z* = 2.04, *p* < 0.05). This paradoxical result fits well with the idea that much of these effects are not causal and thus artifactual. A likely cause of this artifact is that there may be individual differences in sensitivity for the two positions, real or perceived. Thus, participants may be more sensitive to one-side or the other, and this bias should naturally lead to greater choice of the more sensitive side, which will automatically lead to more repetition, necessarily associated with better performance. But, as the repetition is not causal, it should be equally strong for one-forward as for one-back, as we observed. Figure 5F shows that this was indeed the case. Most participants showed a strong bias, more commonly toward the top than the bottom. The maximum bias was about 83% with an average absolute bias of 55%, presumably driving the bias in choice in the shuffled data.

Figure 5G plots the accuracy advantage against the position choice bias across conditions (plotting data of Figure 5E against 5D). Unlike for orientation (Figure 3F), the two were not significantly correlated (*r* = 0.35, *p* = 0.32, logBF = −0.22), showing that position choice bias did not covary with accuracy advantages and therefore bestowed little advantage on the task.

**Figure 6.**
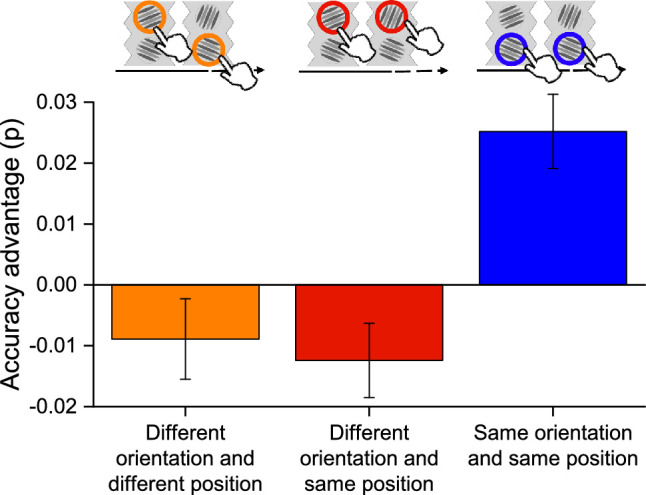
Bar graphs showing the accuracy advantage for the condition where participants chose a stimulus where the previous trial (one-back) was a Gabor patch with different orientation and different position (orange bar), different orientation and the same position (red bar), or the same orientation and same position (blue bar). Error bar represents ±1 *SEM.*

The results described above suggest that there is a strong tendency to choose the previously selected position (stickiness). This can lead to an increase in accuracy, but the increase would seem to be largely artifactual, given that the effects of future stimuli are as strong or stronger than the effects of past stimuli. To examine this effect further, we divided the trials on the basis of the orientation of the previous stimulus. The results show that only those where the previous orientation was the same showed an advantage in accuracy (*Z* > 3.29, *p* < 0.001) (Figure 6).

## Discussion

In this study, we measured SD with a technique designed to study confidence, where participants first chose which of two noisy stimuli they preferred to judge, then made a forced-choice judgment on the orientation of the chosen stimulus. Stimuli with the same orientation as the previous stimulus in that position were chosen more frequently than those of a different orientation, and, more importantly, choosing the same orientation led to better accuracy. The advantage in accuracy for congruent trials persisted for trials up to five-back and accumulated over trials.

SD typically results in a bias in perception, revealed by systematic reproduction errors or other means. Although this has shown to result in more efficient perception, largely by reducing response variance (Cicchini, Arrighi, Cecchetti, Giusti, & Burr, 2012; Cicchini et al., 2014; Cicchini, D'Errico, & Burr, 2022; Jazayeri & Shadlen, 2010), the question as to whether there are benefits in two-alternative, forced-choice paradigms remains open. With standard two-alternative, forced-choice paradigms, serial dependence, which should lead to a response bias, would not increase sensitivity. If the stimuli are truly random, the bias will help on half of the trials but hinder the other half, resulting in no net gain. However, the current study design showed that continuity of orientation can activate automatic and pre-decisional mechanisms, affecting the confidence-based decisions and also improving the accuracy. These results are in line with evidence that SD acts at the level of sensory mechanisms (Cicchini et al., 2014; Cicchini et al., 2017; Cicchini et al., 2018; Fischer & Whitney, 2014) rather than decisional processes (Fritsche et al., 2017). Participants never explicitly chose the stimulus of the same orientation; rather, they chose up or down based on which stimulus they believed had the stronger signal (Barthelmé & Mamassian, 2009). Yet, this choice led to a small preference in choosing the continuous stimulus. The continuous stimuli were also judged more accurately than the discontinuous stimuli. Although the choice of the continuous stimulus was very close to 50% (about 51%), when it was chosen there was a significant improvement in accuracy, by about 2.4%. This suggests that, even though repetition of orientation leads to only a small increase in choice, when the condition is chosen its orientation is seen more accurately. The most probable explanation for the increase in accuracy is that serial dependence boosts signal strength rather than merely biasing perceptual decisions.

The complex paradigm allowed us to look for other types of SD, such as for the position chosen (irrelevant for the task). Here, the results were quite different. There was a very strong bias to choose the previously selected position, by about 10%. However, most of this bias was also presented for contingency with future stimuli and in the shuffled dataset (∼5%), suggesting that it was not driven by SD. There was also an apparent advantage in accuracy, but, again, this advantage disappeared when compared with shuffled results, except for the contingency on the future. The results are probably explained by individual biases, driven by higher sensitivity of superior or inferior visual fields. The biases were strong, up to 83% and 55% on average. The main message from this analysis is that there are many artifacts that can cause the impression of serial dependence, and these artifacts can be stronger than the real effects. In our paradigm, the task was orientation judgment, and orientation was completely independent of position. Despite the strong artifact related to position stickiness, repetition of orientation reliably affected both stimulus choice and response accuracy. This suggests that the effects of serial dependence, both on choice and accuracy, are robust.

Studies in serial dependence typically measure biases induced by successive stimuli differing by small amounts in a key dimension (often orientation). This study used a technique very similar to that usually employed in serial dependence studies, except that it did not measure bias in perceived orientation but rather what may be considered to be a bias in saliency, leading to increased choice and increased accuracy. Nevertheless, we can be quite confident that the bias in choice and the improvement in accuracy are associated with serial dependence rather than other local after-effects such as adaptation. Our stimuli were very similar to those typically used for serial dependence studies: low spatial frequency gratings of moderate contrasts, often noise masked (Cicchini et al., 2017; Cicchini et al., 2018; Cicchini et al., in press; Collins, 2020; Fischer & Whitney, 2014; Murai & Whitney, 2021). Adaptation paradigms typically use long presentations, well over a second, whereas our presentation was 100 ms. Presentation time is a strong predictor of whether the serial effects will be assimilative or repulsive (Cicchini et al., in press; Yoshimoto, Uchida-Ota, & Takeuchi, 2014).

Our study shows that, during a serial dependence paradigm, the sensory representation of the previously presented orientation is effectively boosted. In this respect, the results resemble those of perceptual priming, where repetition of a feature (often color) improves performance, measured as accuracy or reaction times (Maljkovic & Nakayama, 1994). That a typical serial dependence paradigm also leads to improvements suggests that the two processes share common neural mechanisms; however, this suggestion is not supported by other evidence. Galluzzi et al. (2022) reported evidence suggesting that the two are quite distinct, in that there is very little correlation between many important aspects. This does not exclude the possibility that the two processes share some neural circuitry, but current evidence would suggest that they are not identical. Clearly, more research is needed to understand the exact interplay.

To sum up, we present a new paradigm for investigating SD, as it provides a clean technique to dissociate the effects of real SD from artifacts.
